# Tyrosine phosphatase SHP2 aggravates tumor progression and glycolysis by dephosphorylating PKM2 in gastric cancer

**DOI:** 10.1002/mco2.527

**Published:** 2024-04-04

**Authors:** Peiyun Wang, Yueting Han, Wen Pan, Jian Du, Duo Zuo, Yi Ba, Haiyang Zhang

**Affiliations:** ^1^ Tianjin Medical University Cancer Institute and Hospital National Clinical Research Center for Cancer Tianjin's Clinical Research Center for Cancer Key Laboratory of Cancer Prevention and Therapy Tianjin Medical University Tianjin China; ^2^ The Institute of Translational Medicine Tianjin Union Medical Center of Nankai University Tianjin China

**Keywords:** gastric cancer, glycolysis, PKM2, posttranslational modifications, SHP2

## Abstract

Gastric cancer (GC) is among the most lethal human malignancies, yet it remains hampered by challenges in fronter of molecular‐guided targeted therapy to direct clinical treatment strategies. The protein tyrosine phosphatase Src homology 2 domain‐containing phosphatase 2 (SHP2) is involved in the malignant progression of GC. However, the detailed mechanisms of the posttranslational modifications of SHP2 remain poorly understood. Herein, we demonstrated that an allosteric SHP2 inhibitor, SHP099, was able to block tumor proliferation and migration of GC by dephosphorylating the pyruvate kinase M2 type (PKM2) protein. Mechanistically, we found that PKM2 is a bona fide target of SHP2. The dephosphorylation and activation of PKM2 by SHP2 are necessary to exacerbate tumor progression and GC glycolysis. Moreover, we demonstrated a strong correlation between the phosphorylation level of PKM2 and adenosine 5‘‐monophosphate (AMP)‐activated protein kinase (AMPK) in GC cells. Notably, the low phosphorylation expression of AMPK was negatively correlated with activated SHP2. Besides, we proved that cisplatin could activate SHP2 and SHP099 increased sensitivity to cisplatin in GC. Taken together, our results provide evidence that the SHP2/PKM2/AMPK axis exerts a key role in GC progression and glycolysis and could be a viable therapeutic approach for the therapy of GC.

## Graphical abstract

We determined PKM2 as a new SHP2 substrate in GC and identified PKM2‐Y105 as a critical residue that affected the interacted effect. In addition, we unveiled direct regulation of PKM2 Y105 dephosphorylation by SHP2 with the GC glycolysis. Then, the important role of the SHP2/PKM2/AMPK positive feedback axis in the GC process has been verified.

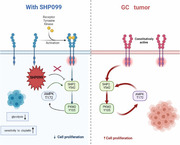

## 1 INTRODUCTION

Gastric cancer (GC) remains a prevalent and aggressive cancer in the world. It causes more than 1,000,000 new cases reported in 2020 and an estimated 769,000 death cases (equivalent to one in every 13 death cases in the world), ranking fifth in global incidence and fourth in mortality.^1^ Recent noteworthy findings are that in both low‐risk and high‐risk areas, the incidence of GC in young adults (aged <50 years) has increased.^2^, ^3^ The trend of younger patients with GC makes it more threatening, so there is an urgent need to find genes and signal pathways that affect the development of GC and excavate potential therapeutic targets.

Posttranslational modifications (PTMs) refer to the covalent modifications after protein biosynthesis that regulate multiple cellular processes. The rapid response of cells to environmental changes is usually controlled by the reversible covalent modifications of existing proteins, which can be reflected in the phosphorylation of protein hydroxyl amino acids Tyr, Ser, and Thr in eukaryotic cells.^4^, ^5^ PTMs (including phosphorylation, ubiquitination, acetylation, methylation, glycosylation, and palmitoylation) have a profound impact on the function of proteins and play a vital role in almost all cellular biological processes. The diversity of PTMs and their crosstalk is related to many key signal events of tumor transformation, carcinogenesis, and metastasis. The pathological effects of various PTMs are involved in tumor marker function, tumor metabolism, and tumor microenvironment regulation.^6^, ^7^, ^8^

Src homology 2 domain‐containing phosphatase 2 (SHP2), encoded by the PTPN11 gene, is a nonreceptor tyrosine phosphatase (PTP), which is a signal‐enhancing transducer, functioning between RTKs and RAS.^9^ Improper regulation of SHP2 is involved in progression of many diseases involving developmental disorders, inflammatory diseases, cancers, and metabolic diseases.^10^, ^11^, ^12^, ^13^ SHP2 is considered to be one of the key hubs linking Cag‐A to GC.^14^, ^15^ Interaction of Cag‐A with SHP2 leads to an imbalance in SHP2 phosphatase activity, which is indispensable for the complete activation of the RAS‐ERK pathway.^16^ Besides, SHP2 and SOS synergistically promote the activation of RAS, and SHP2 combined with MEK inhibition inhibits KRAS‐amplified gastroesophageal cancer cell proliferation.^17^ Due to the important role of SHP2 in cancer, SHP099, a selective, potent, orally bioavailable allosteric inhibitor of SHP2, has attracted considerable attention and has been previously tested in clinical trials in advanced solid tumors.^18^, ^19^ Although the function of SHP2 in cancers has been well elucidated, its role in GC progression and function remains questionable.

Pyruvate kinase M2 type (PKM2) can affect the last step of aerobic glycolysis, which is to catalyze the transfer of phosphate groups from phosphoenolpyruvate (PEP) to adenosine di‐phosphate to generate adenosine tri‐phosphate (ATP) and pyruvate. Therefore, PKM2 promotes the glycolysis of cancer cells. The phosphorylation modifications of PKM2 affect the activity of pyruvate kinase (PK), exert a vital role in cancer glycolysis, and are crucial for the growth of cancer cells.^20^ Of note, tyrosine phosphorylation suppresses PKM2 activity.^21^, ^22^ However, the correlation between the activity of PKM2 and the tumor is intricate and remains controversial.

The purpose of our study was to explore the mechanism of SHP2 promoting the occurrence and development of GC, and to explore the role of SHP2 expression on the malignant characteristics of GC by cell function and mouse tumor‐bearing experiments. Using the results of the shotgun LC–MS analysis, we screened and verified the downstream substrates of SHP2, and analyzed that SHP2 affects the glycolysis of GC by affecting the phosphorylation level of PKM2. Then the important role of the SHP2/PKM2/adenosine 5‘‐monophosphate (AMP)‐activated protein kinase (AMPK) positive feedback axis in the GC process was verified. Finally, the effect of a small‐molecule SHP2 inhibitor—SHP099—on the sensitivity of cisplatin was analyzed, to offer a theoretical basis for the development of SHP099 clinical application.

All in all, in this study, we determined that PKM2 is a new SHP2 substrate in GC and that PKM2‐Y105 is the key residue that affects their interaction. In addition, we also determined the regulatory mechanism of PK activity in GC by studying tyrosine phosphorylation. The important role of SHP2/PKM2/AMPK positive feedback axis in GC process is verified. This positive cascade can be interfered by SHP099, thus hindering the malignant behavior of GC by inhibiting glycolysis of cancer cells. Our results also show that SHP2 may be a potential therapeutic target to improve the sensitivity of GC cells to cisplatin, which provides a theoretical basis for SHP099 combined with cisplatin in the treatment of GC. Our study provides a new idea for the clinical study of SHP2 in GC, which can promote the malignant biological behavior of GC by promoting the glucose metabolism of GC.

## 2 RESULTS

### 2.1 SHP2 is involved in GC pathogenesis

**FIGURE 1:**
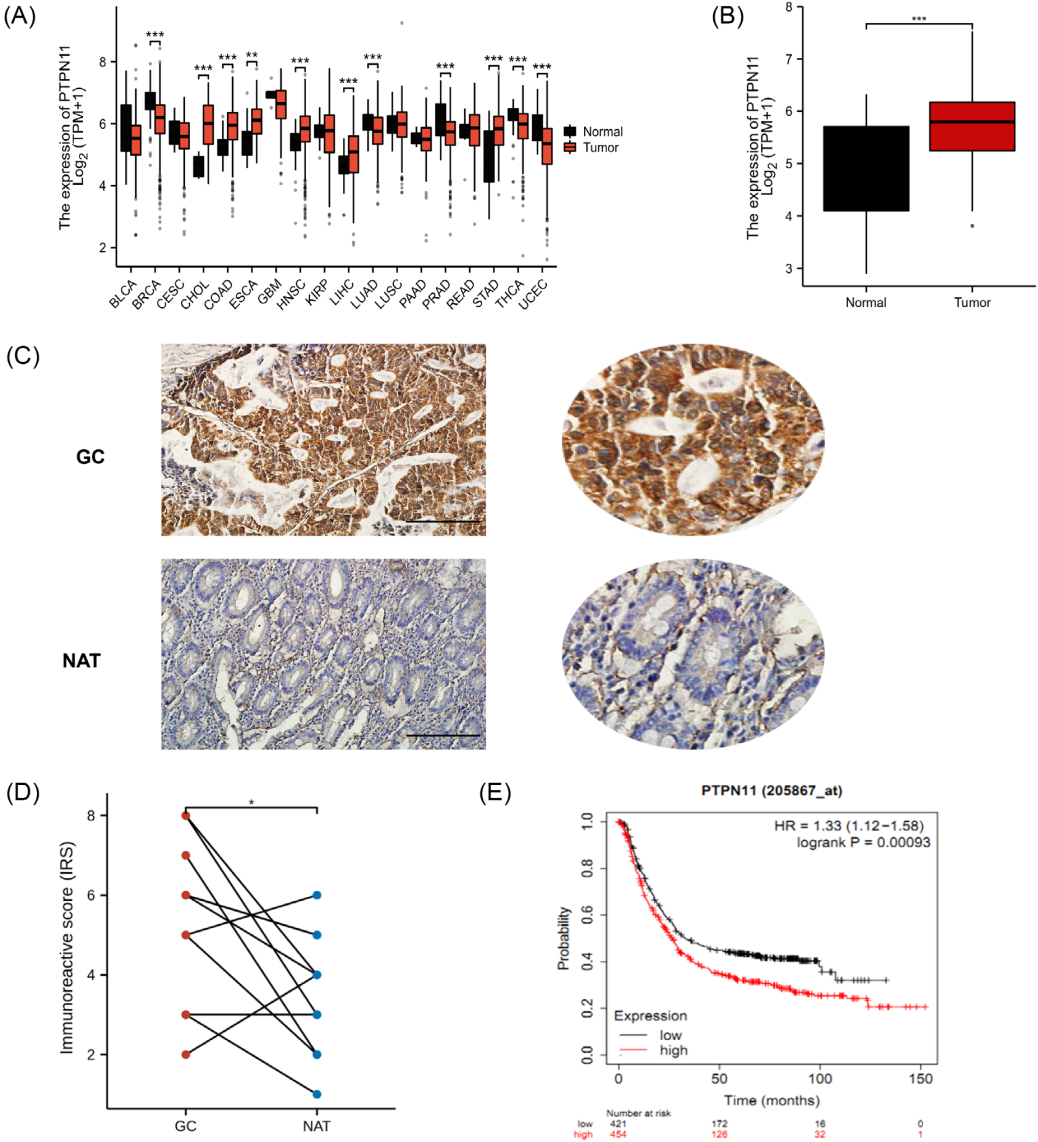
SHP2 was overexpressed in gastric cancer tissues, and indicated a poor prognosis. (A) The expression level of PTPN11, a gene encoding SHP2, in different tumors. (B) The expression level of SHP2 in gastric cancer tissues. (C) SHP2 expression in GC and normal tissues adjacent to the tumor (NAT) was detected by immunohistochemistry. scale bar = 500 µm. (D) The immunoreactive score of (C) (*n* = 12 pairs). (E) The correlation between SHP2 expression and overall survival in GC was counted with the log‐rank test. *p < 0.05; **p < 0.01; and ***p < 0.001.

To clarify the expression levels of SHP2 in GC, we analyzed the The Cancer Genome Atlas (TCGA) database and found that SHP2 was diffusely expressed in multiple tumors, including stomach cancer (Figure 1A), which indicating that SHP2 may be participated in the vicious transformation a variety of tumors including GC. TCGA analysis found that the PTPN11 is highly expressed in GC (Figure 1B). To explore the relationship between SHP2 and clinical characteristics, we examined the expression distribution of SHP2 in GC tissues by immunohistochemistry (IHC). As shown in Figures 1C and D, SHP2 expression in GC tissues was obviously higher than that in para cancer. The characteristic of patients was shown in Table S1. In addition, we analyzed the effect of SHP2 on the prognosis of GC by Kaplan–Meier plotter, survival analysis showed that GC patients who with higher SHP2 expression lever indicated a worse prognosis (Figure 1E). Overall, the above results show that phosphatase SHP2 is abnormally highly expressed in GC and indicates adverse outcomes.

### 2.2 SHP2 promotes the proliferation and migration of GC cells

**FIGURE 2:**
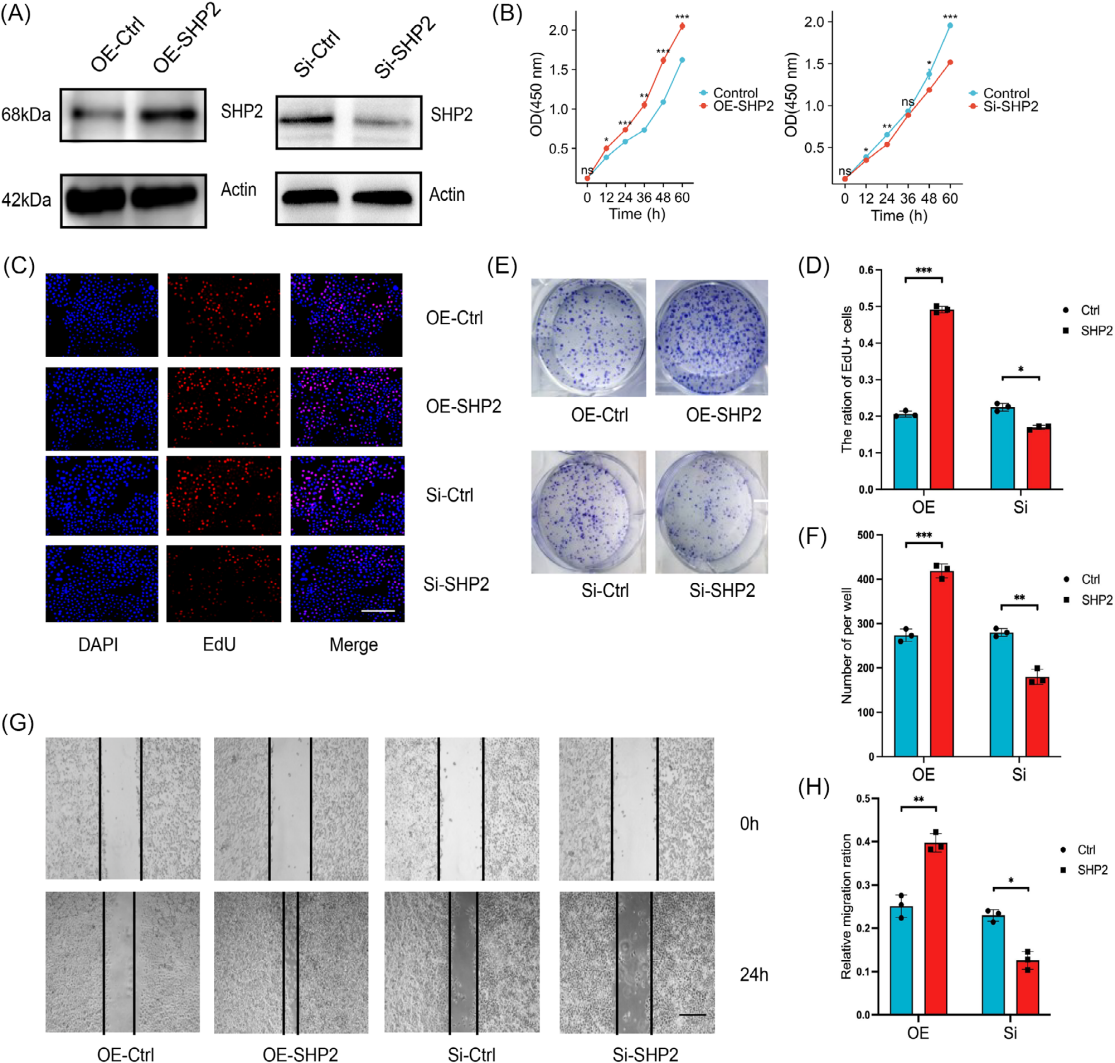
The relationship between SHP2 and malignant biological behavior in gastric cancer. (A) WB analysis of the expression of SHP2 in HGC‐27 cells in the different treated groups. (B) The CCK8 proliferation assay of HGC‐27 cells with SHP2 overexpression or silence. (C) The HGC‐27 cells were stained with EdU, and then the EdU positive cells were counted to evaluate the proliferation of GC cells. scale bar = 200 µm. (D) Quantitative analysis of (C) (*n* = 3). (E) The effect of SHP2 expression level on the proliferation in HGC‐27 cells was determined by clone formation assay. (F) Quantitative analysis of (E) (*n* = 3). (G) The migratory capacity of HGC‐27 cells was tested in a scratch assay. scale bar = 50 µm. (H) Quantitative analysis of (I) (*n* = 3). *p < 0.05; **p < 0.01; and ***p < 0.001.

To determine whether SHP2 affects the biological characteristics of GC cells, we conducted proliferate and migrate experiments to assess. We used Western blotting (WB) to show the effect of overexpression and knocking down of SHP2 (Figure 2A). Compared with the control group, a significantly higher absorbance value at 450 nm was observed in the OE‐SHP2 group. The growth rate of the SHP2 downexpression group was remarkably lower than that of the control group (Figure 2B). Besides, EdU assays revealed that the downexpression of SHP2 considerably impeded the proliferation rate of GC cells in vitro (Figures 2C and D). As shown in Figures 2E and F, the ability of colony formation was weakened in the si‐SHP2 GC cells. The scratch assays were employed to measure the role of SHP2 on the migration function of GC cells. Decreased expression of SHP2 resulted in a considerable decrease of the migration ability of GC cells (Figures 2G and H). Taken together, the in vitro data convincingly show that SHP2 promotes proliferate and migrate ability to involve in GC progression.

### 2.3 SHP2 interacted with PKM2 and combined PKM2 at the Y105 site

**FIGURE 3:**
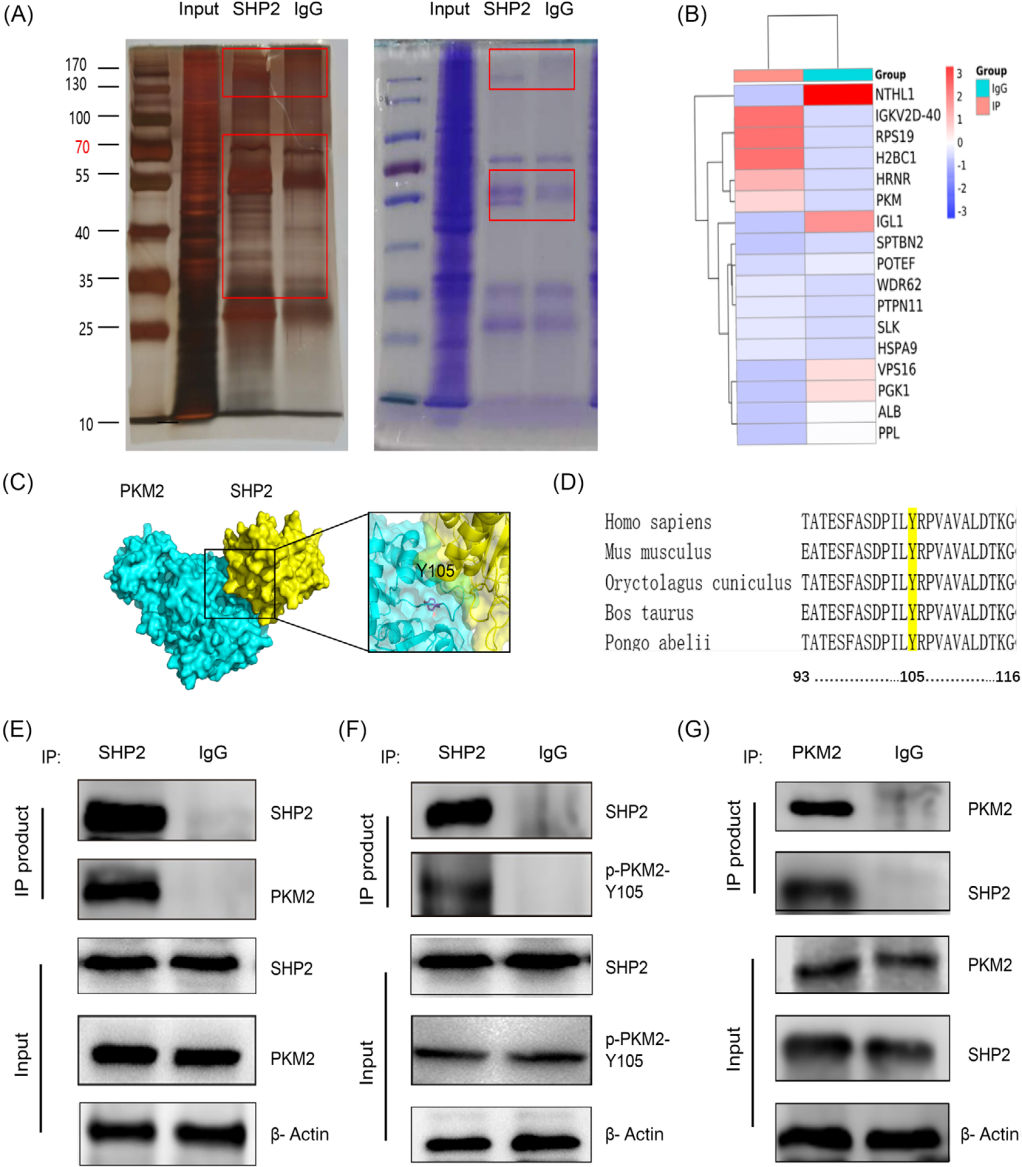
Tyr 105 site of PKM2 interacts with SHP2 in GC cells. (A) Immunoprecipitation (IP) was revealed by SDS‐PAGE‐silver staining or Coomassie brilliant blue staining. (B) The IP‐coupled shotgun LC–MS of the protein product screened the proteins that may bind to SHP2. (C) We adopted HADDOCK 2.4 to perform the protein–protein docking.^48^ Protein docking showed the possibility and potential binding sites of SHP2 and PKM2 binding. (D). Sequence comparison reveals a quite pronounced across the sequence conservation in PKM2‐Y105. (E–G) Cell lysate from HGC‐27 cells were incubated with SHP2 antibody (Ab) or PKM2 Ab or IgG, and then subjected to WB analysis with SHP2 Ab, PKM2 Ab, or p‐PKM2‐Y105 Ab.

To determine the SHP2 substrate(s) of GC, a variety of discovery‐driven methods were adopted. The SHP2 binding proteins in GC were immunoprecipitated by anti‐SHP2 antibody. Silver staining and Coomassie Blue staining were performed after electrophoresis (Figure 3A). Differential protein samples between irrelevant IgG and anti‐SHP2 antibodies were analyzed by shotgun LC–MS. MS results identified 10 proteins binding with SHP2 (Figure 3B and Table S2). Since PKM2 has higher unique peptides, its protein size position is in the position of the staining difference band, and its relationship with SHP2 has not been clarified, we choose PKM2 as the potential substrate of SHP2. Protein docking found that SHP2 and PKM2 have the possibility of binding and PKM2‐Y105 is a potential combined site (Figure 3C). In addition, sequence alignment of the PKM2 of multiple species indicated that the Tyr‐105 site is conserved among the detected species, suggesting that the Tyr‐105 site is evolutionarily conserved (Figure 3D). Immunoprecipitation of SHP2 in HGC‐27 cells further indicated that SHP2 interacted with PKM2 (Figure 3E). Immunoprecipitation of SHP2 further indicated that SHP2 interacted with PKM2‐Y105 (Figure 3F). Similarly, enriching PKM2 by anti‐PKM2 antibody pulled down not only PKM2 but also SHP2, which further proves that they are combined (Figure 3G). Taken together, we have discovered the new substrate of SHP2, which proves that SHP2 and PKM2 combined to each other in GC cells, and PKM2‐Y105 is a potential binding site.

### 2.4 SHP2 dephosphorylated PKM2 and affected the glycolysis level of GC by binding to PKM2

**FIGURE 4:**
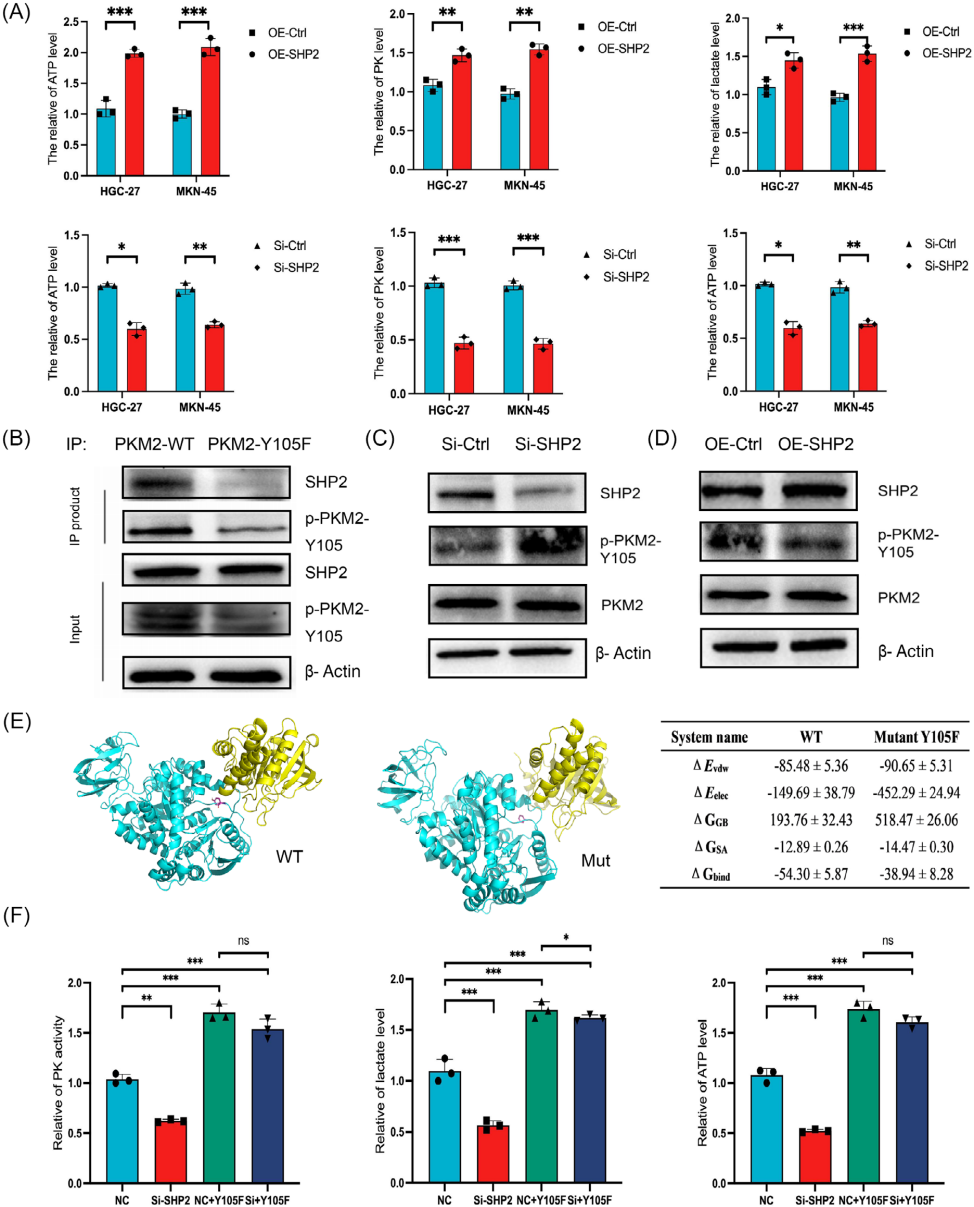
Effect of SHP2 on glycolysis of GC by dephosphorylation of PKM2. (A) The relative of lactate production, PK activity and ATP level in different treated groups in HGC‐27 and MKN‐45 cells. (B) Co‐IP analysis of the interaction between SHP2 and wild‐type PKM2 (PKM2‐WT) or dephosphorylation mimic mutants (PKM2‐Y105F) in HGC‐27 cells. (C and D) The phosphorylation level at Y105 site of PKM2 was altered by SHP2 overexpression or silence in HGC‐27 cells. (E) Molecular dynamics (MD) simulations indicated that the Tyr105 mutation impaired the flexibility of the substrate‐binding region. The red mark in the picture represents 105 residues. Δ*E*_vdW_: van der Waals energy. Δ*E*_elec_: electrostatic energy. Δ*G*_GB_: electrostatic contribution to solvation. Δ*G*_SA_: nonpolar contribution to solvation. Δ*G*_bind_: binding free energy. (F) The relative of lactate production, PK activity and ATP level were tested in rescued assay in MKN‐45 cells. *p < 0.05; **p < 0.01; and ***p < 0.001.

Since PKM2 catalyzed the final step in the cancer glycolysis, it was rational to reckon that the interactions between SHP2 and the glycolytic enzyme would affect the glycolytic metabolism process in GC. To determine the difference in cell metabolism in different groups, we used the corresponding kits to detect the PK activity, lactate level, and ATP production of cells. Overexpression of SHP2 was shown to facilitate glycolysis by boosting PK activity, lactate level, and ATP production (Figure 4A). The identified Tyr (Y) sites were mutated to Phe (F) to deny phosphorylation and mimic dephosphorylation. The co‐immunoprecipitation (Co‐IP) results indicated that the Tyr105 mutation impairs the binding ability with SHP2 (Figure 4B). WB analysis showed that the PKM2 Y105 phosphorylation expressions enhanced in the Si‐SHP2 group, while total PKM2 expression levels remained little changed (Figure 4C). In contrast, an increase in SHP2 level via transfection with SHP2 overexpression plasmids reduced the level of PKM2 phosphorylation (Figure 4D). In order to illustrate the nature of the complex interactions, molecular docking, and molecular dynamics (MD) simulation were undertaken. The kinetics of candidate mutants are detected by simulation. The simulations indicated that the Tyr105 mutation impairs the flexibility of the substrate‐binding region. The binding energy of WT was higher than that of mutants, indicating that the mutation would destroy their binding (Figure 4E). The induced PK activity, lactate production and ATP lever resulting from SHP2 downexpression were reversed by a constitutively dephosphorylated mutant PKM2 (Figure 4F), establishing a functional link between SHP2 and PKM2 in promoting cancer glycolysis. The results showed that SHP2 affects the glycolysis of GC by affecting the phosphorylation level of PKM2.

### 2.5 SHP2‐mediated dephosphorylation of PKM2 affected AMPKα phosphorylation levels in GC cells

**FIGURE 5:**
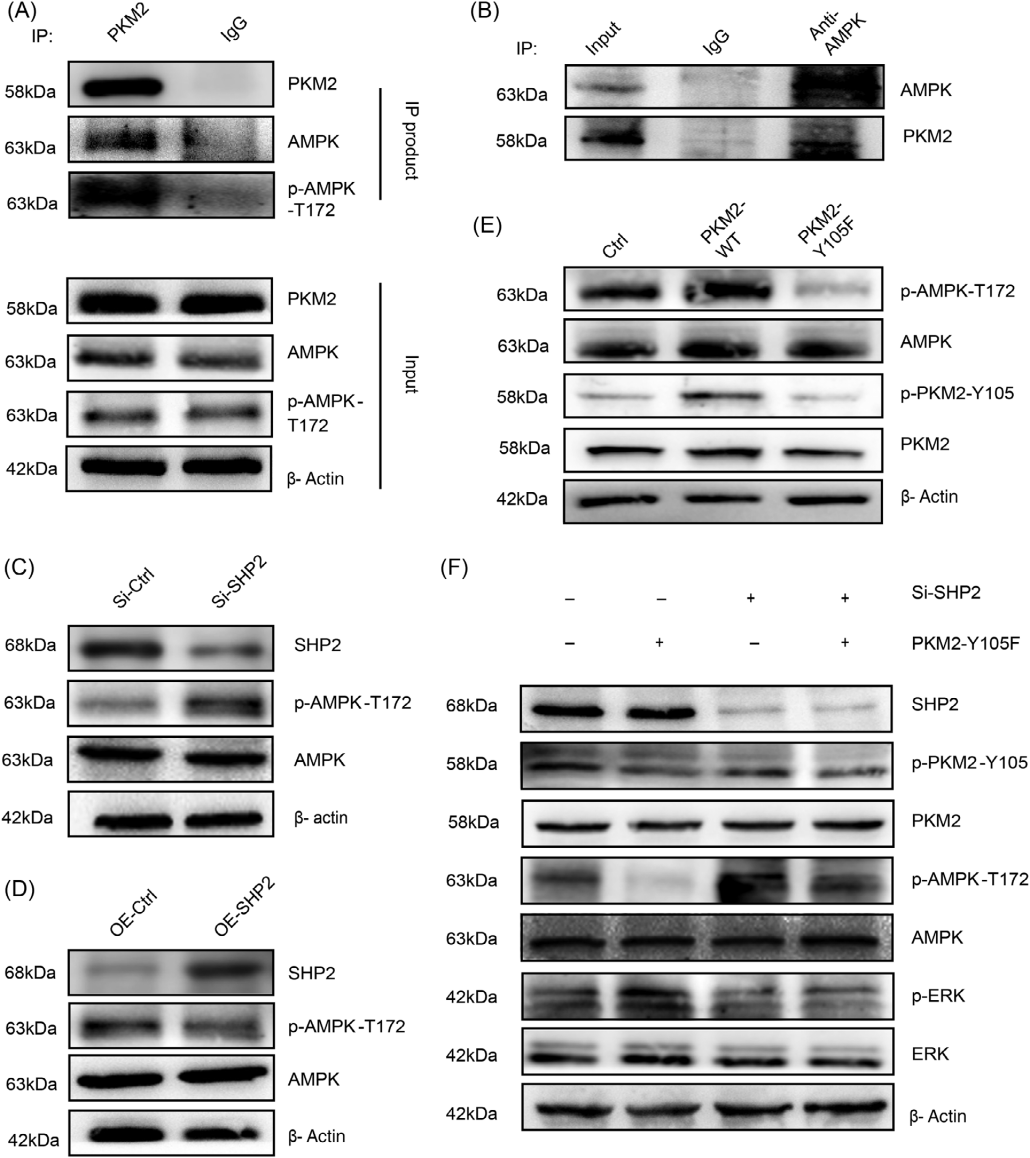
SHP2‐mediated dephosphorylation of PKM2‐Y105 altered AMPKα‐T172 phosphorylation levels in GC cells. (A and B) Co‐IP analysis of the interaction between PKM2 and AMPK or p‐AMPK‐T172 in MKN‐45 cells. (C and D) The phosphorylation level at T172 site of AMPK was altered by SHP2 overexpression or silence in MKN‐45 cells. (E) The dephosphorylation of PKM2‐Y105 affected AMPKα phosphorylation levels in MKN‐45 cells. (F) SHP2‐mediated dephosphorylation of PKM2 altered AMPKα phosphorylation levels in MKN‐45 cells.

AMPKα and p‐AMPKα were immunoprecipitated by anti‐PKM2 antibody (Figure 5A). As expected, AMPKα was immunoprecipitated, and the immunoprecipitation samples were probed for PKM2 by immunoblotting (Figure 5B). WB analysis showed that the phosphorylated level of AMPKα at the T172 in the Si‐SHP2 group was significantly increased, while the background levels of AMPKα remained little changed (Figure 5C). Overexpression of SHP2 significantly decreased the phosphorylation level of AMPKα and SHP2 overexpression little change in total levels of AMPKα (Figure 5D). As shown in Figure 5E, compared with the PKM2‐WT, the phosphorylation expression of PKM2 and AMPKα in the PKM2‐Y105F were remarkably decreased. These results suggest that PKM2 interacts with AMPKα directly and affects AMPKα phosphorylation. Interestingly, the phosphorylation of AMPK was obviously induced after the additional expression of PKM2‐Y105F in SHP2‐down expressed cells, with the levels of AMPK total protein expression rarely changed (Figure 5F). In summary, SHP2‐mediated dephosphorylation of PKM2 at Y105 affects the level of AMPKα phosphorylation at T172 site in GC cells.

### 2.6 SHP099 impeded the growth and migration of GC cell in a dose‐dependent fashion and SHP2/PKM2/AMPK formed a positive feedback loop in GC

**FIGURE 6:**
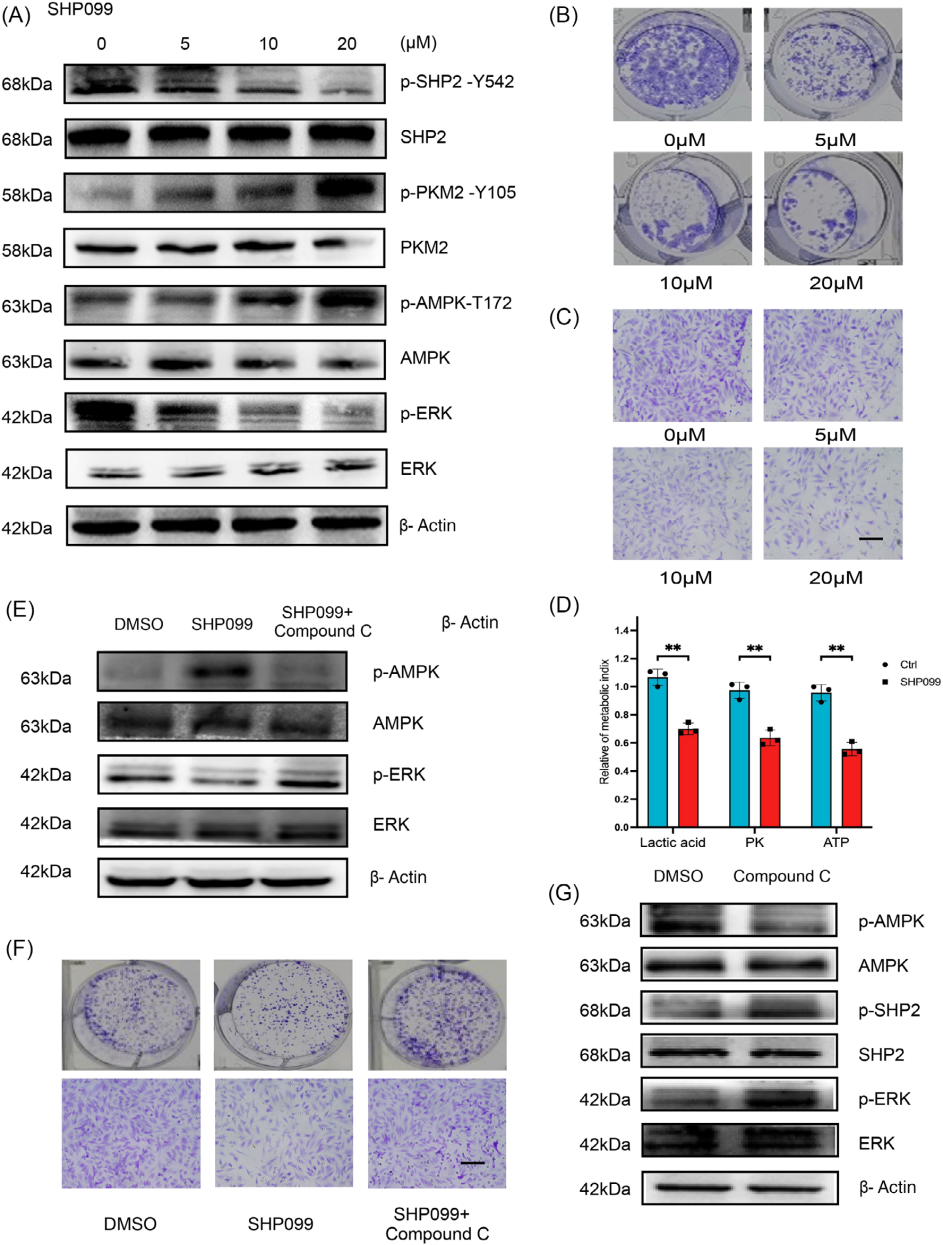
SHP099 suppressed the proliferation and migration of GC cells, and SHP2/PKM2/AMPK forms a positive feedback loop in GC. (A) The effect of different doses of SHP099 on the phosphorylation level of SHP2, PKM2, AMPK, and ERK in MKN‐45 cells. (B and C) Effects of SHP099 at different concentrations on the clonal forming ability and migratory ability of HGC‐27 cells. (D) The influence of SHP099 on different metabolic indexes in MKN‐45 cells. (E) Compound C reversed the effect of SHP099 on AMPK phosphorylation level in MKN‐45 cells. (F) Compound C reversed the effect of SHP099 on migration ability of HGC‐27 cells. (G) Compound C activated SHP2 was verified by WB in HGC‐27 cells. **p < 0.01.

SHP099 is the allosteric inhibitor of SHP2, which can stabilize the self‐inhibitory closed conformation of SHP2, thereby suppressing its phosphatase activity. We next assessed the antitumor efficacy of SHP099 in vitro. WB results indicated that with the treatment of SHP099, the protein expression of p‐SHP2 and p‐ERK weakened, while the expression of p‐PKM2 and p‐AMPK rose in a dose‐dependent manner (Figure 6A). SHP099 dose dependently blocked the migrated and proliferated ability in GC cells (Figures 6B and C). The metabolic index of PK activity, lactic acid level, and ATP level were decreased under the effect of SHP099 (Figure 6D). Compound C is a cell‐permeable AMPK inhibitor, which decreases AMPK phosphorylation.^23^, ^24^ Combined SHP099+Compound C treatment reduced phosphorylation levels of AMPK and promoted phosphorylation levels of ERK (Figure 6E). As expected, the combination of SHP099 and Compound C could reverse the resistance to migration of SHP099 (Figure 6F). Surprisingly, the WB results showed that Compound C could activate SHP2 (Figure 6G). Above all, these data suggested that SHP2/PKM2/AMPK formed a positive feedback loop in GC.

### 2.7 Cisplatin activated SHP2 and SHP099 increased sensitivity to cisplatin

**FIGURE 7:**
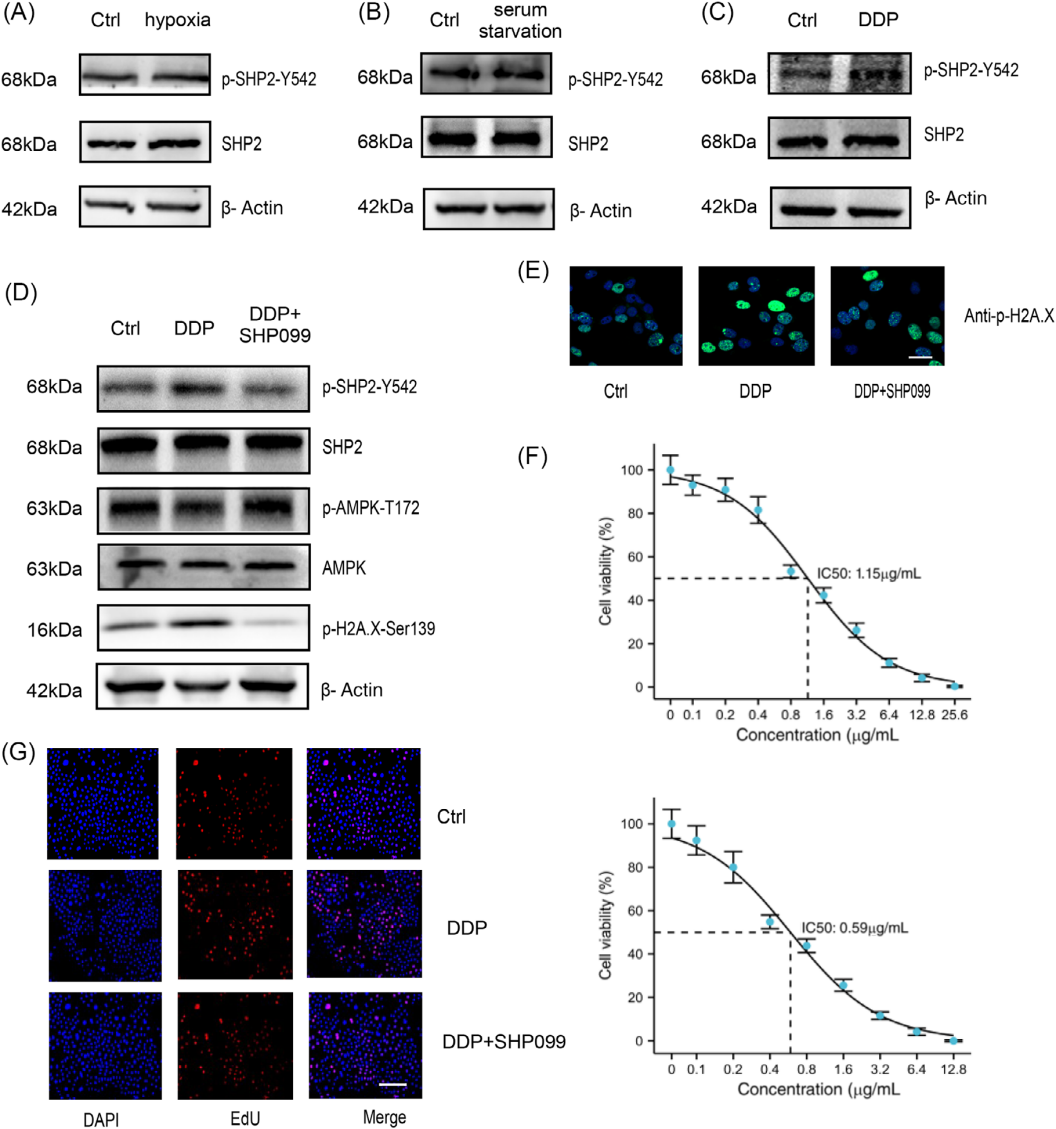
DDP activated SHP2 in GC cells. (A–C) The influence of different stimuli on the activation state of SHP2 in HGC‐27 cells. (D) WB analysis of the phosphorylation expression of SHP2, AMPK, H2A in HGC‐27 cells. (E) γH2A.X staining in the indicated groups was imaged via immunofluorescence in HGC‐27 cells. (F) The DDP+SHP099 group had the higher sensitivity displaying lower half maximal inhibitory concentration (IC50) than DDP alone group in GC cells by CCK8 assay. (G) Evaluation of the rate of proliferating cells after treated with DDP or DDP+SHP099 by EdU assay. Scale bar = 200 µm.

In order to study the factors involved in activating SHP2, several experiments were carried out. Only DDP significantly trigger activation of SHP2 (Figure 7A). While other stimuli such as hypoxic treatment and nutrient starvation hardly activate SHP2 (Figures 7B and C). The phosphorylated histone H2A.X (aka γ‐H2A.X) offers a platform for anchoring DNA damage repair factors to patch DNA damage.^25^ To assess the levels and distribution of γ‐H2A after combinatorial SHP099+DDP treatment, both WB and immunofluorescence were performed. The combined use of SHP099 and DDP could reverse the activation of SHP2 induced by DDP and reduce the level of H2A phosphorylation (Figure 7D). In addition, immunofluorescence staining analysis showed similar results. Compared with cisplatin alone, the immunofluorescence intensities of γ‐H2A reversed in combinatorial SHP099+DDP treatment (Figure 7E). As shown in Figure 7F, the inactivation of SHP2 resulted in a decrease in the IC50 (greater sensitivity) to DDP in GC. EdU assay indicated that a combination of DDP and SHP099 could further hamper the growth ability of GC (Figure 7G). Therefore, these results suggested that coordinated suppression of H2A contributes to increased apoptosis after combinatorial SHP099+DDP treatment. Taken together, the above data suggested that increased activation of SHP2 is a characteristic of DDP resistance in GC and that inhibition of SHP2 may be a feasible treatment proposal for reversing the drug resistance to DDP.

### 2.8 The in vivo role of SHP2 in aggravating GC progression through SHP2/PKM2/AMPK axis

**FIGURE 8:**
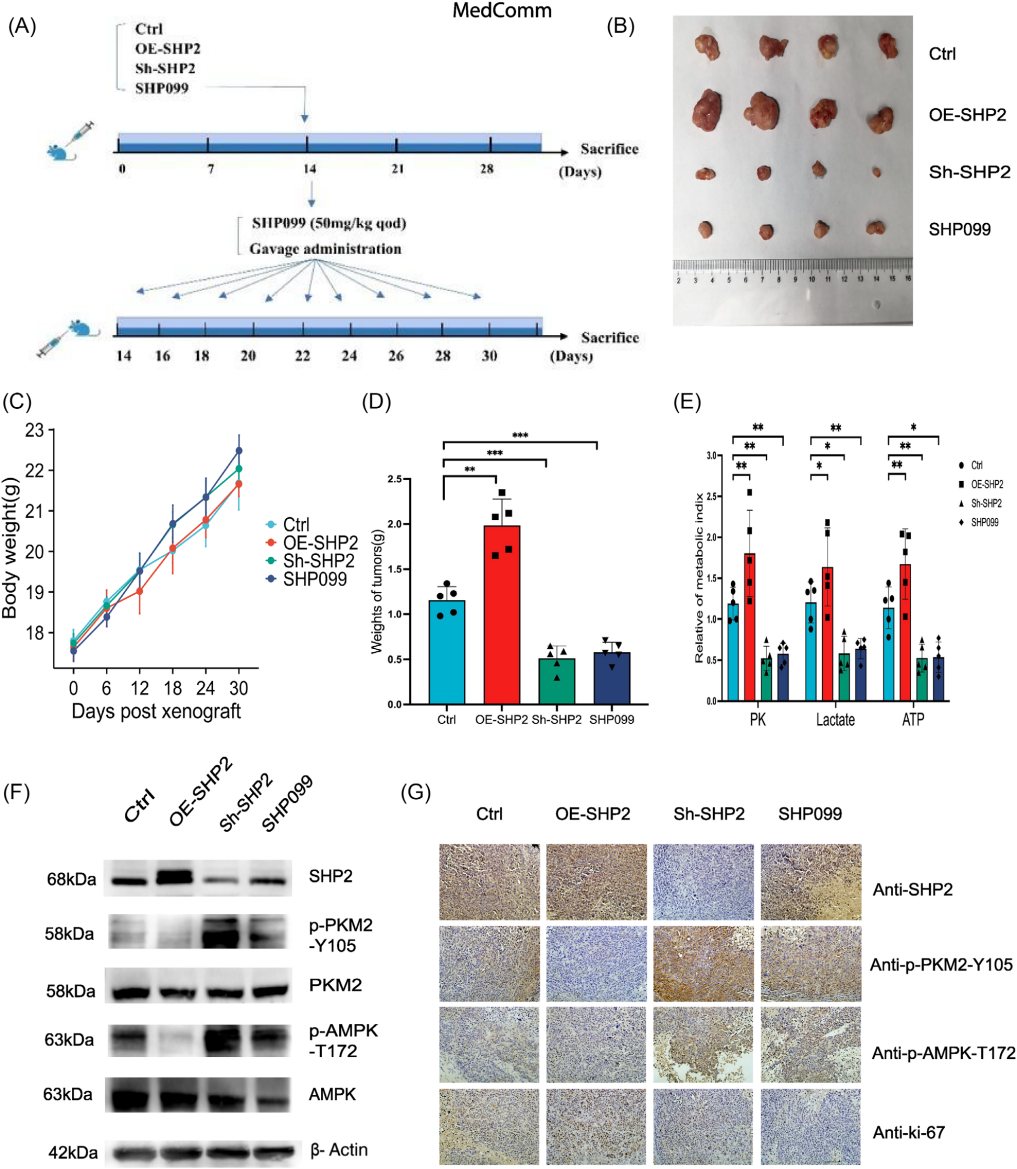
In vivo validation for SHP2/PKM2/AMPK positive feedback loop. (A) The flow charts design for in vivo experiments. The model of mice was created with BioRender.com. (B) Morphology and size of xenografted tumors in mice after sacrifice. (C) The body weight of mice in different groups. (D) Quantitative analysis the weight of tumors (*n* = 5). (E) The quantitative analysis of metabolic index of tumor tissues in different groups. (F) WB quantification of the expression of SHP2, p‐PKM2, and p‐AMPK in tumors tissues in the different groups. (G) The expression of SHP2, p‐PKM2, p‐AMPK, and Ki‐67 in xenografted tumors tissues obtained from the different groups was evaluated by IHC. *p < 0.05; **p < 0.01; and ***p < 0.001.

To explore the function of SHP2 on GC progression in vivo, mouse xenograft models were established. 50 mg/kg of SHP099 and each mice was administered 100 µL methylcellulose by intragastric gavage have been selected to establish xenograft model.^17^ The flow chart illustrated the specific grouping and administration methods (Figure 8A). SHP2 overexpression showed a growing in tumor diameter and volume. Similarly, SHP099 suppressed transplanted tumor to grow (Figure 8B). Judging from the changes of body weight in different groups, there were no obvious toxic and side effects in the administration group. (Figure 8C). This conclusion was identified with the result of tumors weight (Figure 8D). Next, we tested the metabolic index of nude mouse tissues. Specifically, lactic acid content, PK activity, and ATP content were increased in tumors with overexpressed SHP2 (Figure 8E). WB further determined the expression of SHP2, phosphorylated PKM2 and AMPK in different xenograft tumor tissues (Figure 8F). IHC results indicated that SHP2 overexpression enhanced cells proliferation rate in tumor tissues, and SHP2 expression was negatively correlated with the phosphorylated PKM2 and AMPK (Figure 8G). The results in vivo indicated that OE‐SHP2 could improve the glycolysis in nude mice possibly by decreasing the phosphorylation level of PKM2 and AMPK.

In this study, we determined PKM2 as a new SHP2 substrate in GC and identified PKM2‐Y105 as a critical residue that affected the interacted effect. Besides, we determined the regulatory mechanism of PK activity in GC through tyrosine phosphorylation. In addition, we unveiled direct regulation of PKM2 Y105 dephosphorylation by SHP2 with the GC glycolysis. Then, the important role of the SHP2/PKM2/AMPK positive feedback axis in the GC process has been verified. This positive cascade can be interfered by SHP099, thereby hindering the GC process by regulating cancer cells glycolysis (Graphical Abstracts). (Adapted from “FLT3 Mutations in Acute Myeloid Leukemia (AML)”, by BioRender.com (2020). Retrieved from https://app.biorender.com/biorender‐templates.)

## 3 DISCUSSION

Although recent advances in diagnosis and treatment strategy of GC have perfected clinical outcomes, feasible treatments are still lacking in many GC patients.^26^, ^27^ Globally, GC is associated with a large number of cancer‐related deaths^28^; increasing our understanding of the pathological process in this tumor is essential for the diagnosis and therapy strategies of GC.

Under physiological and pathophysiological conditions, the paradoxical effect of protein tyrosine phosphatase and tyrosine kinase regulates signal transduction mediated by phosphorylated tyrosine.^29^, ^30^, ^31^ Understanding the dynamic regulation of phosphorylated tyrosine signal in GC may provide a way for the therapeutic intervention of GC.

The SHP2 protein embodies two SH2 structures (namely N‐SH2 and C‐SH2), which play a key part in the subcellular localization of SHP2, and the PTP domain plays a key role in its enzymatic activity.^32^ SHP2 is involved in various malignant biological behavior related to tumor development, such as metastasis, drug resistance, and tumor microenvironment remodeling.^33^, ^34^, ^35^ Recently, various allosteric inhibitors with significant inactivating effects against SHP2 have been identified.^36^, ^37^ A variety of evidence suggests that SHP2 promotes tumor initiation and development in GC patients.^15^, ^17^, ^38^, ^39^ Hence, identifying the new substrate that regulated by SHP2 may promote to the development of improved antineoplastic therapies. In our findings, we proposed that the inhibition of PKM2 activity by SHP099 hinders GC cells’ glycolysis and the malignant process by disrupting the phosphorylation levels of PKM2.

Since it was discovered that PKM2 is a tyrosine‐binding protein,^40^ interests in the modification of the tyrosine site of PKM2 has increased. The interaction of phosphotyrosine peptide with PKM2 results in the release of the allosteric activator fructose‐1,6‐diphosphate, thereby inhibiting the enzymatic activity of PKM2.^20^, ^40^, ^41^ When PKM2 is allosterically suppressed, its catalytic activity is below than the upstream glycolysis rate; therefore, PEP and upstream glycolysis intermediates could amass and flux into the anabolic pathway; conversely, when PKM2 is allosteric activated, its activity is beyond the upstream glycolysis rate, the upstream glycolysis intermediate would stream into pyruvate, which is turned into lactic acid or enters oxidation.^42^, ^43^, ^44^ Studies have indicated that the receptor tyrosine kinase FGFR1 phosphorylates the PKM2 Y105 to inactivate its activity and affect the Warburg effect.^45^ PKM2‐Y105 is activated by kinase phosphorylation to exert oncogenic function, partly by activating YAP downstream signal transduction to increase the characteristics of cancer stem cell‐like cells in breast cancer cells.^46^ Notably, recent study has supported that the dephosphorylation of PKM2‐Y105 fostered tumor growth in pancreatic cancer.^47^

The results show that SHP2 served as a promising therapeutic target to enhance the sensitivity of GC cells to cisplatin, offering a theoretical foundation for the combined therapies of SHP099 and cisplatin in GC. In general, these findings provide a new insight into the dephosphorylation of PKM2 by SHP2 and its metabolic regulation in GC. However, our study still lacks the verification of a large number of clinical samples, and the effect of SHP2 on the nuclear translocation of PKM2 in the process of PKM2 phosphorylation needs to be further demonstrated in future studies.

## 4 MATERIALS (OR SUBJECTS) AND METHODS

### 4.1 Human samples

We obtained human GC and relative tissues adjacent to carcinoma from patients who were undergoing surgery at the Tianjin Medical University Cancer Institute and Hospital (Tianjin, China). Written informed consent was obtained from all participants. Ethical approval was granted by the Ethics Committee of Tianjin Medical University Cancer Institute and Hospital (Approval number: Ek2021002).

### 4.2 Animals

For animal experimentation, female BALB/c‐nu mice (4 weeks of age) were purchased from the GemPharmatech Co., Ltd. (Nanjing, China) and housed in a special pathogenic‐free animal facility. The operational process was executed within the approve of the Institutional Animal Care and Research Advisory Committee of Tianjin Medical University Cancer Institute and Hospital (Approval number: NSFC‐AE‐ 2021374).

### 4.3 Cell culture

The human GC cell line HGC‐27 and MKN‐45 were acquired from the cell bank of the Chinese Academy of Sciences (Shanghai, China). The cells were cultured in in RPMI 1640 culture medium (Gibco, Grand Island, NY, USA), which was supplemented with 10% fetal bovine serum (FBS) (BI) and 1% penicillin/streptomycin (Gibco). All cell culture were grown in carbon dioxide cell incubator at 37% with 5% CO_2_. All cells have been authenticated by STR profiling.

### 4.4 Cell transfection

Si‐SHP2 and negative controls were obtained from RiboBio Co., LTD (Guangzhou, China). The sequence of si‐SHP2 was GGTCCAGTATTACATGGAA. Cells were transfected with Lipofectamine 2000 (Invitrogen, Carlsbad, CA, USA) in accordance with the manufacturer's instructions. Overexpressed and control plasmids of SHP2, WT‐PKM2 and mut‐PKM2 plasmids were obtained from Jikai Gene Chemical Technology Co. Ltd. (Shanghai, China).

### 4.5 Protein extraction and WB

Cells and tissues protein were extracted with lysis buffer supplemented with fresh protease and phosphatase inhibitors. The total lysate was separated by SDS‐PAGE gel. Then transferred to cut polyvinylidene fluoride membranes (Roche, Basel, Switzerland). Membranes blocking were carried out using 5% bovine serum albumin (BSA). The relative antibody to incubating membranes at 4°C with overnight. Chemiluminescence reaction was taken by an enhanced chemiluminescence (ECL) reagent (EpiZyme Scientific, Shanghai, China) after incubated with the suitable secondary antibody at room temperature for 1 h. The information of antibodies were shown in Table S3.

### 4.6 IHC assay

The tissues were fixed and embedded in paraffin. After deparaffinization, antigen retrieval, peroxidase removal, and blocking, the tissues were incubated with the corresponding antibodies overnight. Then all sections were stained with DAB system (ZSGB‐BIO; Beijing, China) and counter‐stained with hematoxylin (Biosharp, Anhui, China). Five random regions of the per specimen were analyzed under the microscope. The staining intensity score of tissue was 0 for no staining, 1 for weak staining, 2 for moderate staining, and 3 for strong staining. The staining percentage of specimens was evaluated at the same time: 0–25% scored 1 point, >25–50% scored 2 points, >50–75% scored 3 points, >75–100% scored 4 points. The immunohistochemical score (immunoreactivity score) was multiplied by the intensity and the staining percentage, and the score was 0−12.

### 4.7 Co‐immunoprecipitation

The cell lysate was incubated with relative antibody or IgG (Santa Cruz) overnight at 4°C. Then the mix coincubated with Protein A/G PLUS‐Agarose beads (Santa Cruz). The protein complexes were separated via SDS–PAGE, and the gels were stained with silver (Beyotime, Shanghai, China) and Coomassie (EpiZyme Scientific), then analyzed via shotgun LC–MS/MS by Genechem Co., Ltd (Shanghai, China).

### 4.8 Cell proliferation assay

(1) Cell Counting Kit (CCK‐8) (Biosharp) was adopted to detect cell proliferate ability. Cells (1000 cells in the Figure 2 and 5000 cells in the Figure 7) were seeded in 96‐well plates, and after treatment for 48 h, the medium was replaced with new culture medium supplementing with 10% CCK‐8. The cells were then incubated at incubator for 2 h. Then, the optical density value at 450 nm wavelength was measured on the microplate reader to assess the degree of yellow color in the medium.

(2) The EdU assay was detected by EdU proliferation kit (Ribo‐Bio). Briefly, after treatment for 48 h, cells were incubated with 50 µM EdU for suitable time. Then fixation, permeabilization, and EdU staining were undertaken following manufacturer's instructions. Subsequently, cell nuclei were stained with Hoechst 33342. Finally, the results were photographed by a Leica microscope and the proportion of EdU‐positive cells was examined.

### 4.9 Clone formation assay

The cells were seeded in six‐well plate (500 or 1000 cells/well). When clones could be seen by naked eyes, fixed them with methanol:glacial acetic acid (3:1) fixative (15 min). After rinsing three times with PBS, the cells were stained with 0.1% crystal violet solution for 15 min.

### 4.10 Cell migration assay

The cell migratory ability was assessed by transwell chambers (Costar). About 1 × 10^5^ cells were seeded into the upper chambers with 200 µL of serum‐free culture media, while 600 µL of medium containing 20% FBS was added to the lower chamber. After 12 h, the migrated cells were fixed and stained.

### 4.11 PK activity and lactate product assay

Assay of relative PK activity was followed the manufacturer's protocol of Pyruvate Kinase Activity Assay Kit (Nanjingjiancheng, Nanjing, China). The lactate levels were detected through a Lactate Assay Kit (Nanjingjiancheng). Enzyme activities and lactate production were normalized on the basis of cell numbers or protein contents.

### 4.12 ATP measurement

Cells were seeded into opaque 96‐well plates. A CellTiter‐Glo Luminescent Cell Viability Assay (Promega, Madison, WI, USA) was adopted to ATP measurement. The pretreated cells were stored at room temperature for half an hour. Next, 100 µL of CellTiter‐Glo reagent was added to each well, followed by mixing for 2 min on a shaker and incubation at room temperature for 10 min according to instructor's protocol. Then the luminescence was assessed by a microplate reader.

### 4.13 Protein‐docking and MD simulations

Protein–protein docking was performed using HADDOCK 2.4. MD simulations were performed using AMBER18. MM/GBSA calculations were performed based on trajectories obtained by MD. Binding free energies and energy components were predicted by MM/GBSA.

### 4.14 Immunofluorescence

The cells were seeded into 24‐well plates with cell climbing slices. The cells were fixed with 4% fixative solution and permeated with Triton X‐100. After 1 h of blocked with 5% BSA at RT, incubated with relative antibodies overnight at 4°C. Then suitable second antibody (ZSGB‐BIO) was incubated at RT for 1 h and nuclear was stained with DAPI (Sigma).

### 4.15 Establishment of mouse tumor‐bearing model

MKN‐45 cells were relatively transfected with lentivirus to overexpress or downexpression of SHP2, and untreated MKN‐45 cells were used as a control. 5 × 10^6^ MKN‐45 cells were subcutaneously injected into each mouse to establish the GC xenograft model. When the tumors grew to a suitable size, a unit of 50 mg/kg of SHP099 (Selleck Chemicals) was administered by oral gavage. Each mice was administered 100 µL methylcellulose by intragastric gavage.

### 4.16 Statistical analyses

The data were derived from at least three independent experiments and were showed as the mean ± SE. Difference between the group were calculated by an unpaired Student's *t*‐test and analysis of variance. *p* < 0.05 recognized statistically significant. *p < 0.05; **p < 0.01; and ***p < 0.001.

## AUTHOR CONTRIBUTIONS

P. W., J. D., and W. P. performed most of the experiments, and wrote the manuscript. Y. H. analyzed the data. P. W. and D. Z. reviewed and edited the manuscript. Y. B. and H. Z. designed the experiments and edited the manuscript. H. Z. is the guarantor of this work, had full access to all data reported in the study, and takes responsibility for the integrity of the data and the accuracy of the data analysis. All authors read and approved the final paper.

## CONFLICT OF INTEREST STATEMENT

The authors declare that they have no competing interests.

## ETHICS STATEMENT

The research was approved by the Ethics Committee of Tianjin Medical University Cancer Institute and Hospital. The study was performed in accordance with the Declaration of Helsinki. All of the authors agree to the submission and consent for publication this paper. Approval number for animal: NSFC‐AE‐2021374. Approval number for clinical experiment: Ek2021002.

## Supporting information

Supporting information

## Data Availability

The data that support the findings of this study are available from the corresponding author upon reasonable request.
