# Supplementary material for: Tyrosine phosphatase SHP2 aggravates tumor progression and glycolysis by dephosphorylating PKM2 in gastric cancer
Source: MedComm (2020). 2024 Apr 4;5(4):e527. doi: 10.1002/mco2.527 (PMC10993348; doi:10.1002/mco2.527)
Supplement: Supplementary file 1 — Supporting information [file MCO2-5-e527-s001.docx]

**Supplementary material**

**Tyrosine phosphatase SHP2** **aggravates tumor progression and glycolysis by dephosphorylating PKM2 in gastric cancer**

Peiyun Wang^1,#^, Yueting Han^1,#^, Wen Pan^1,#^, Jian Du^1,#^,Duo Zuo^1,#^, Yi Ba^1,*^ , Haiyang Zhang^1,2,*^

・Authors’ affiliations:

^1^Tianjin Medical University Cancer Institute and Hospital, National Clinical Research Center for Cancer, Tianjin’s Clinical Research Center for Cancer, Key Laboratory of Cancer Prevention and Therapy, Tianjin Medical University, Tianjin, 300060, China

^2^The Institute of Translational Medicine, Tianjin Union Medical Center of Nankai University, Tianjin 300121, China.

^#^ Equal contributors

^*^Joint corresponding authors

・Corresponding author:

Haiyang Zhang ([zhanghaiyang@tmu.edu.cn](mailto:zhanghaiyang@tmu.edu.cn);) and Yi Ba ([bayi@tjmuch.com](mailto:bayi@tjmuch.com)). The Full Postal address: Tianjin Medical University Cancer Institute and Hospital, Huan hu xi Road 18, Tianjin 300060, China.

**Table S1** The characteristic of patients for IHC.

| **Characteristics** | **Number of patients ( % ) n=12** |
| --- | --- |
| Age, median (range)  Sex  Female  Male  Smoking history  Yes  No  Family history  Yes  No  KPS status  90-100  70-80  Tumor location  Gastric antrum  Gastric body  Tumour grade  Well differentiation  Medium differentiation  Low differentiation  Metastasis  Yes  No | 58 (46-76)  5 (41.67)  7 (58.33)  5 (41.67)  7 (58.33)  3 (25)  9 (75)  8 (66.67)  4 (33.33)  10 (83.33)  2 (16.67)  0 (0)  4 (33.33)  8 (66.67)  8 (66.67)  4 (33.33) |

**Tabe S2**  The details of MS results.


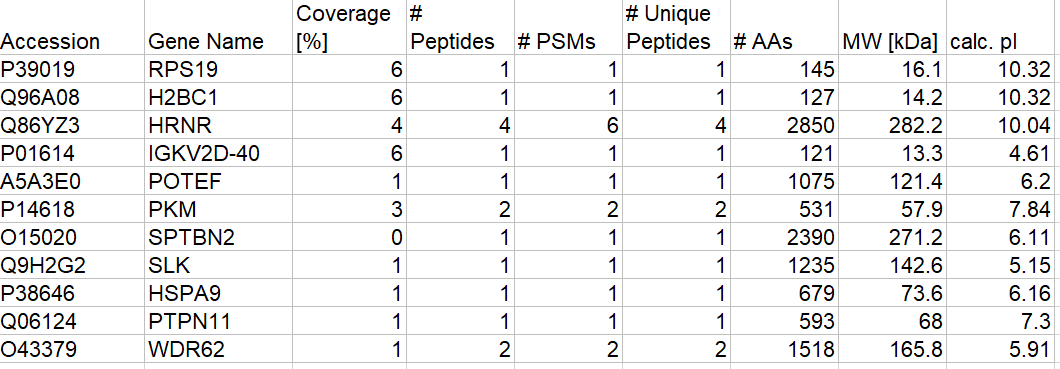


**Tabe S3** The information of antibodies for WB.

| Antibody | Dilution Rate |
| --- | --- |
| SHP2 (Santa Cruz)  p-SHP2(Y542) (Santa Cruz)  PKM2 (Santa Cruz)  p-PKM2(Y105) (ImmunoWay)  AMPK (Santa Cruz)  p-AMPK(T172) (Cell Signaling Technology)  ERK (Santa Cruz)  p-ERK (T202/Y204) (Santa Cruz)  β-actin (Bioss)  goat-anti-rabbit IgG (Solarbio)  goat-anti-mouse IgG (Solarbio) | 1:1000  1:1000  1:1000  1:2000  1:1000  1:1000  1:1000  1:1000  1:3000  1:5000  1:5000 |
